# Incorporating Carbon Nanotubes in Nanocomposite Mixed-Matrix Membranes for Gas Separation: A Review

**DOI:** 10.3390/membranes12060589

**Published:** 2022-05-31

**Authors:** Aimi Farzana Yazid, Hilmi Mukhtar, Rizwan Nasir, Dzeti Farhah Mohshim

**Affiliations:** 1 Department of Chemical Engineering, Universiti Teknologi PETRONAS, Sri Iskandar 32610, Malaysia; hilmi_mukhtar@utp.edu.my; 2 Department of Chemical Engineering, University of Jeddah, Afsan Road, Jeddah 23890, Saudi Arabia; rnasir@uj.edu.sa; 3 Department of Petroleum Engineering, Universiti Teknologi PETRONAS, Sri Iskandar 32610, Malaysia; dzetifarhah.mohshim@utp.edu.my

**Keywords:** mixed-matrix membranes, carbon nanotubes (CNTs), nanocomposite, gas separation, membrane technology

## Abstract

Carbon nanotube (CNT) is a prominent material for gas separation due to its inherent smoothness of walls, allowing rapid transport of gases compared to other inorganic fillers. It also possesses high mechanical strength, enabling membranes to operate at high pressure. Although it has superior properties compared to other inorganic fillers, preparation of CNTs into a polymer matrix remains challenging due to the strong van der Waals forces of CNTs, which lead to agglomeration of CNTs. To utilize the full potential of CNTs, proper dispersion of CNTs must be addressed. In this paper, methods to improve the dispersion of CNTs using functionalization methods were discussed. Fabrication techniques for CNT mixed-matrix membrane (MMM) nanocomposites and their impact on gas separation performance were compared. This paper also reviewed the applications and potential of CNT MMMs in gas separation.

## 1. Introduction

In recent years, membrane technologies have become prominent and have been used in gas processing, such as nitrogen gas separation from the air, purification of natural gas, and the removal of hydrogen in different petrochemical processes and refinery streams [1]. Further separation technologies, such as absorption, adsorption, and cryogenic, do not offer the same benefits as membrane technologies [2]. The membrane has a non-complex operation system that is easy to handle with high energy efficiency, leading to low capital and operating cost. There are many interests in technology as the world is shifting towards green technology. In 2019, the global market for membrane technology was valued at $10.88 billion, up from $7.02 billion in 2014, showing the growth of interest over the years [3]. However, there are still many ways that this technology can be improved. The advantages and limitations of current separation technology are highlighted in Table 1.

Relative to other membrane applications, studies on gas separation membranes have been the center of interest in the oil and gas manufacturing and chemical industries. Hydrogen/nitrogen separation from ammonia plants, nitrogen removal from air, hydrogen/hydrocarbon separations, and natural gas recovery are some of the membrane technologies used in gas separation industries. The concept of membranes in gas separation was first proposed by Thomas Graham in 1866 when he suggested using an academic paper made of polymers to separate gas mixtures [9]. This concept became a reality when Loeb and Sourirajan produced a high-flux asymmetric membrane composed of cellulose acetate for reverse osmosis, which was subsequently used in gas separation [3,4]. However, the technology was only first brought to a large industrial scale by Permea (Air Product) in 1980 for hydrogen separation. Afterward, the development of membrane technology for industrial scales escalated and bloomed exponentially. Researchers since then have been focusing on improving membrane properties in chemical and mechanical structures, configurations, and applications [10,11]. 

Permeability and selectivity are two factors that influence membrane gas separation performance. The permeability coefficient, pressure, and thickness normalized flux provide a quantitative measure of the transport flux of a gas component through a membrane. Membranes with high permeability can handle many gases with a small membrane surface. Selectivity is a measure of separation efficiency represented as the ratio of single gas permeances of any two species penetrating through the membrane. Membranes with high permeability and selectivity are desired, but the Robeson upper bound has a trade-off relationship [12].

Polymer membranes have been widely used in industrial applications and have shown significant improvements over the last two decades. They are more economical than other membranes due to their flexibility and solution processability [13]. However, the performance of polymer membranes in terms of permeability and selectivity is still below the trade-off trend suggested by Robeson. Inorganic membranes show impressive performance, and Koros and coworkers proved this in 1996 by using a carbon molecular sieve (CMS) membrane in gas separation. The result is beyond Robeson’s upper bound [14]. However, the economic factor hindered the membrane due to high fabrication costs and difficulty in fabrication. Furthermore, the membranes are prone to fractures and gaps, are innately brittle, and are challenging to develop into a large surface area module [15].

**Figure 1:**
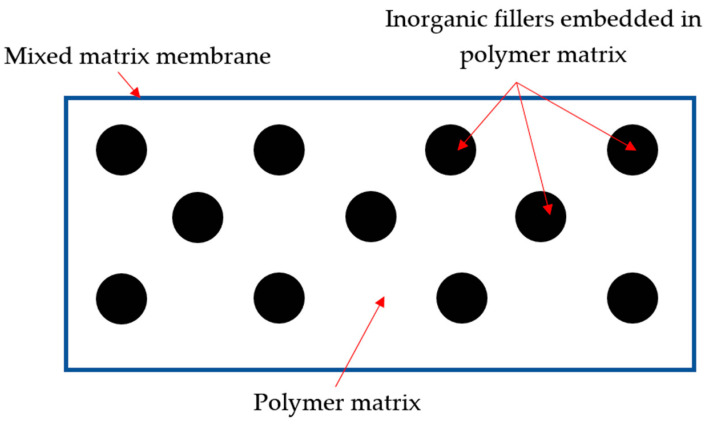
An illustration of an inorganic filler incorporated in a polymer matrix. Reprinted/adapted with permission from Aroon et al. [18] (2010) Elsevier Copyright.

The mixed-matrix membrane (MMM) was developed to circumvent the limitations of polymer and inorganic membranes. It incorporates inorganic fillers into the polymer matrix (as in Figure 1) to increase the volume fraction, increase diffusivity, and create a barrier, thus restricting undesired permeation and improving permeability and selectivity [16]. These hybrid membranes are projected to have higher gas separation performance than pure polymer membranes by combining the desired features of both inorganic and organic phases while being cost-effective for consumers [17]. Inorganic materials used in MMMs improve membranes’ mechanical strength and chemical surface depending on the types of fillers used [18]. Various inorganic fillers have been widely discovered in MMMs, such as metallic organic framework (MOF), carbon molecular sieves (CMS), alumina, zeolite, and carbon nanotubes (CNTs). CNTs are one of the most attractive as they have strong mechanical properties and smoothness that enhance gas’s rapid transport mechanism [19]. CNTs also have fine attributes such as an excellent aspect ratio, nanoscale diameter with superior stability, and efficiency compared to other inorganic materials (which will further be discussed in Section 5). Therefore, MMMs incorporated with CNTs can be used in various gas separation applications. In this paper, methods to improve the dispersion of CNTs are discussed. Fabrication techniques for CNT MMM nanocomposites and their impact on gas separation performance are compared. Moreover, the applications of CNT MMMs in gas separation are also discussed.

## 2. Polymeric Membranes

Polymeric membranes are widely used to introduce membrane technology for large-scale industries. Polymers are used to fabricate membranes for gas separation applications due to their ease of fabrication and can be scaled up for industrial applications. The various polymeric membranes have been commercialized to date, such as the polaris membrane (MTR), cellulose acetate (UOP), cellulose triacetate (Cynara, NATCO Group, Dallas, TX, USA), and polyimides (UBE) [20]. However, Sanders et al. highlighted some of the polymer membranes’ challenges and limitations, such as physical aging, permeability-selectivity trade-off, and plasticization [21].

**Figure 2:**
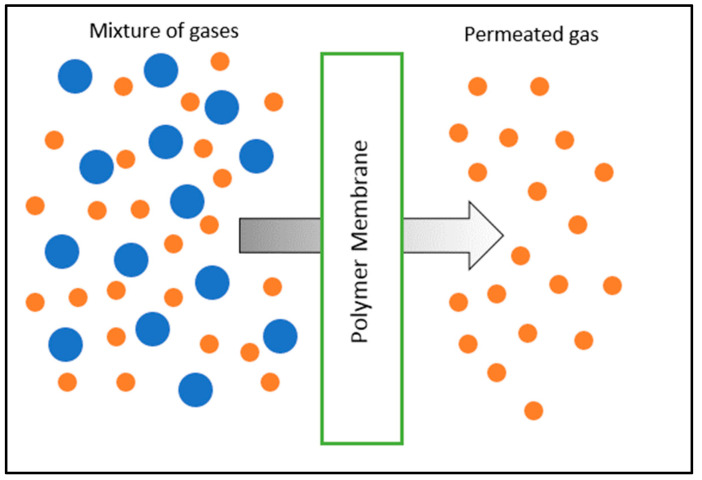
Permeation of gases across polymeric membrane. Reprinted/adaptedwith permission from Khaki et al. [42] (2021) Elsevier Copyright.

Polymers can be categorized into glassy polymers and rubbery polymers. The temperature at which the thermal expansion coefficient changes from a rubbery to a glassy state denotes the boundary between glassy and rubbery polymers and is denoted as the glass transition temperature (T_g_). Rubbery polymers are characterized by recovering their original shape after being strained or distorted [22]. Rubbery polymers have higher diffusivity and permeability than glassy polymers because of their elastic features, including the ability to stretch the chains apart. Nonetheless, this also makes rubbery polymers poor in selectivity performance. Conversely, the glassy polymer is a non-porous material with small, free-volume elements due to its rigid structure resulting from unbending chain rotation [23]. Glassy polymers do not permit long-range chain movements that are feasible in rubbery polymers. It appears to have more segmental movements and more size and shape selection, resulting in better selectivity. The glassy polymer has received substantial recognition because of its mechanical qualities and relatively low production costs [24]. Glassy polymers having tiny free volumes, such as polyethersulfone (PES), polysulfone (PSF), polyimide (PI), and polyetherimide (PEI), have been widely employed in separation membrane processes because of this. Characteristics of glassy and rubbery polymers are shown in Table 2. Moreover, permeation of gases across polymeric membranes is visualized in Figure 2

Fabricating membranes can be divided into a few methods such as phase inversion, stretching, track-etching, and electrospinning [25]. The process for fabricating polymer membranes is determined by the polymer used and the membrane structure required. Liang et al. [26] reviewed the various fabrication techniques and discussed the challenges of fabricating polymeric membranes. Richard et al. [27] reviewed the applications of various polymeric membranes for natural gas processing.

Cartel et al. [28] demonstrated the performance of pristine Matrimid 5218 with four different gases, which are H_2_, N_2_, CO_2_, and CH_4_. The solution-diffusion model can describe the transport of gases through membranes comprising glassy polymers such as Matrimid. In this model, gas molecules initially dissolve into the membrane material at a concentration proportional to the experimental feed-side conditions. Once diffused, gas molecules permeate through the membrane toward the side of the membrane with a lower permeate concentration. According to the literature, the permeabilities of gases are based on the kinetic diameter of gas molecules, as demonstrated in this experiment where H_2_ had the maximum permeability with 30.3 barrer, followed by N_2_ (9.54), CO_2_ (0.70), and CH_4_ (0.32). Mohamed et al. [29] focused on the effect of pressure on the permeability of pure gases (H_2_, CO_2_, N_2_, and CH_4_) at 1–6 bar at 20 °C. The permeability of all gases increased at higher pressure due to the increased driving force for the rapid transport of gases across the membranes. As the pressure increased, the macromolecular segments moved closer to one another. Consequently, the inter-segmental void space diminished, the selected layer area expanded, and the density of the polymer increased, resulting in a rise in permeability.

The critical challenge in polymer membranes is the trade-off between permeability and selectivity, as reported by Robeson in 1991 [30]. A polymer with a novel structure has been the focal interest in increasing separation performance. The existing membrane materials have been modified to minimize the risk, cost, and development duration. Polymer blending is one technique of modifying current membrane materials. By overcoming the limitations of the individual components, polymer blend alteration integrates the synergistic qualities of diverse materials into a new composite with optimized performance [31]. In polymer blends, miscibility between the two combining polymers and solvent is one of the most important factors affecting the membranes’ performance, thermal, and mechanical strength; it is observed by T_g_ and a solubility parameter calculation. A polymer blend is considered homogeneously miscible if a single T_g_ value is obtained; however, two or more Tg events can be observed in a phase-separated polymer blend. Manan et al. [32] reported on PSF/PES blended membranes that demonstrated a 65% increase in ideal selectivity for CO_2_ separation. The blended membranes were thermally stable and could operate at elevated temperatures and pressures. The PSF/PES blend was miscible in all of the compositions, with hydrogen bonding occurring most likely. CO_2_ and CH_4_ gas permeation followed the typical glassy polymer behavior of decreasing permeability and increasing selectivity with increasing pressure.

**Table 2:** Characteristics of Glassy and Rubbery Polymer Materials.

Type of Polymer	Polymer Materials	Characteristics	Limitation	Refs.
Glassy Polymers	PSF	- high plasticization resistance (up to 50 bar) - good thermal, mechanical, and stability properties - excellent in separating CO_2_/CH_4_ because of its similar structure to sulfonyl groups	- moderate separation performance	[30,31]
	Polyimide	- Low mobility of the polymer chain - Superior permeability/selectivity trade-off - High chemical resistance and thermal stability - High mechanical strength - Possesses intrinsic properties due to its imide structure and rigid aromatic moieties	- Has a high degree of polymer chain rigidity, resulting in strong intermolecular interactions - Poor economic viability - Ageing and plasticization issues for long-term uses	[33]
	Cellulose acetate	- Low cost - Ease of processability - Good fouling resistance - High CO_2_ solubility	- Low permeance	[34]
	PES	- Low cost - Long-term thermal stability chemical, and mechanical properties - The polymer’s ether unit provides an alternative mechanism for CO_2_ molecules to bind.	- Moderate plasticization resistance (around 28 bar) - Low permeance	[30,35]
Rubbery Polymers	Pebax	- High mechanical strength and flexibility - Favorable selectivity for acid gas treatment and polar–nonpolar gases such CO_2_/CH_4_ - Increased CO_2_ permeability as a result of the PEO segment’s high affinity for the polar CO_2_ molecule - Has high chain mobility, which results in good interaction with fillers	- Low selectivity	[33,34,35]
	Polyvinyl acetate (PVAc)	- Low cost - Has a strong affinity for CO_2_ and can result in a high solubility of CO_2_ as a result of the polar groups of acetate in its backbone	- Low gas permeance compared to another rubbery polymer - Difficult processability	[36]
	Polyethylene glycol (PEG)	- Due to the high quadruple moment of CO_2_ and the dipole moment of polar ether segments, this material exhibits good CO_2_ permeation characteristics.	- Poor mechanical and thermal properties	[37]
	Polydimethylsiloxane (PDMS)	- Possesses a dense cross-linked network structure and great chain mobility - Low material cost, high thermal and chemical stability	- Favors greater gas transport	[38]

Saeed et al. [39] reported polymer blend membranes to consist of PES and commercial polyetherimide sulfone polymer (Extem) for CO_2_/N_2_ gas separation. Dimethylacetamide (DMAc) was chosen as a solvent due to its good interaction with the polymers, which can facilitate polymer mixing. The permeability of membrane blends significantly increased, whereas the selectivity decreased considerably due to the plasticization effect of high CO_2_ sorption. The membrane surpassed Robeson’s upper limit with a permeability of 6 barrer and selectivity of more than 190 [39]. Elisa et al. [40] prepared a polymer blend of 50/50 wt.% between Matrimid 5218 and a polymer of intrinsic microporosity PIM-EA(H_2_)-TB for CO_2_/CH_4_ gas separation. The permeability performance increased compared to pristine Matrimid while maintaining reasonably high selectivity. The addition of rubbery polymer into membranes generally decreases the selectivity due to the improved mobility chain but is still within the acceptable range.

Rajati et al. [41] reported the performance of Matrimid as a glassy polymer with PVDF as a rubbery polymer. The miscibility of both polymers was achieved at 1–3 wt.% loading of PVDF. The membrane with 3 wt.% PVDF loading had the highest permeability increase of 29% compared to pristine Matrimid. Table 3 presents more studies on gas separation conducted for polymer membranes, highlighting the permeability and selectivity trade-off in such membranes.

## 3. Inorganic Membranes

Inorganic membranes are classified according to their structure: porous or non-porous. Due to a thin top layer that supports the metal, porous inorganic membranes have high mechanical strength and a low mass transfer resistance [46]. They exhibit exceptional stability and durability at elevated temperatures, under harsh impurity, and in hydrothermal environments. Additionally, inorganic membranes are chemically stable and have significantly higher gas fluxes and selectivity [22,47]. These intriguing characteristics have prompted numerous researchers to research the development of inorganic membranes. Inorganic membranes such as alumina [48,49], carbon [50,51], a metal–organic framework (MOF) [52,53], and zeolite [54,55,56] have been rapidly developed and have demonstrated exceptional potential for gas separation applications. Table 4 summarizes the characteristics of each inorganic membrane.

The interest in using inorganic materials for membranes began in 1983, when Koresh and Soffer synthesized the first defect-free hollow fiber CMS membranes via pyrolysis of hollow cellulose fibers and demonstrated separation performance for He, CO_2_, O_2_, and N_2_ [57]. Since then, interest in this membrane has grown due to its molecular sieving properties, resulting in higher selectivity and greater thermal and chemical stability than polymeric membranes [58]. Lin Li et al. [59] discussed the effects of recent hybridized nanoparticle selection on the characteristics and performance of CMS membranes.

**Figure 3:**
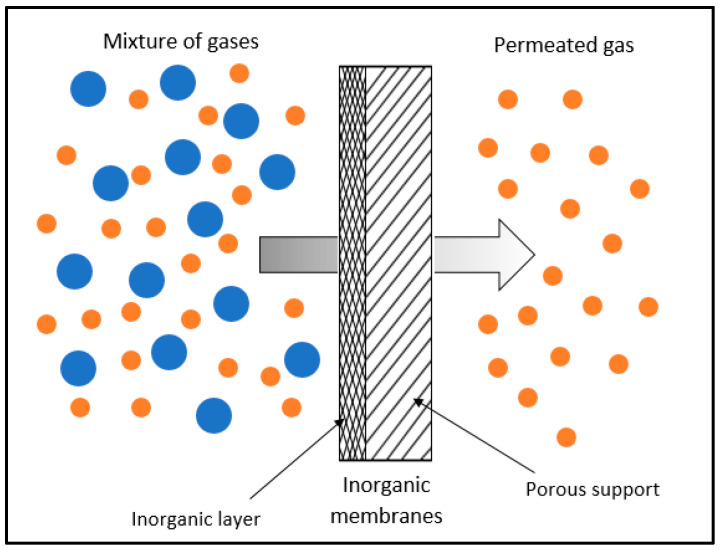
Permeation of gases across inorganic membrane. Reprinted/adapted from Feng et al. [60] © 2022 MPRL.

Although inorganic membranes have been proven to have superior performance and exceed Robeson’s upper bound, the application of inorganic membranes for large-scale industries is not feasible due to the expensive fabrication. Therefore, mixed-matrix membranes have been explored to avoid the disadvantages of individual properties of the polymer and inorganic membranes. Figure 3 illustrates the structure of inorganic membranes.

**Table 4:** Characteristics of inorganic materials.

Inorganic Fillers	Characteristics	Refs.
Zeolite	- Excellent mechanical and thermal stability, as well as resistance to chemicals - Separate gases based on their kinetic diameters - Enhanced separation at lower temperatures due to preferential adsorption	[60,61]
Carbon Molecular Sieve	- High CO_2_/CH_4_ selectivity - Better affinity to glassy polymer	[62]
Graphene Nanosheets	- Large interfacial area - High degree of hydrophilicity - Interlayer spacing between the GNs sheets can be adjusted to optimize the transport of specific molecules. - high flexibility and mechanical strength	[29]
MOF	- High CO_2_ adsorption capacities - Great mechanical flexibility and structure tunability - Synthesized easily and rapidly at a low cost	[53]
Carbon Nanotubes	- Excellent mechanical strength - Inherent smoothness of MWCNTs, which allows rapid transport of gases	[63]
Alumina	- Economical and easily obtainable - Toxic-free substance with a high degree of resistance	[37]

## 4. Mixed-Matrix Membranes (MMMs)

A mixed-matrix membrane is the dispersion of inorganic fillers at the nanometer scale within a polymer matrix to potentially resolve the trade-off relationship between permeability and selectivity [47]. The addition of inorganic filler to the polymeric matrix improves gas diffusion and strengthens the physicochemical properties of MMMs.

**Figure 4:**
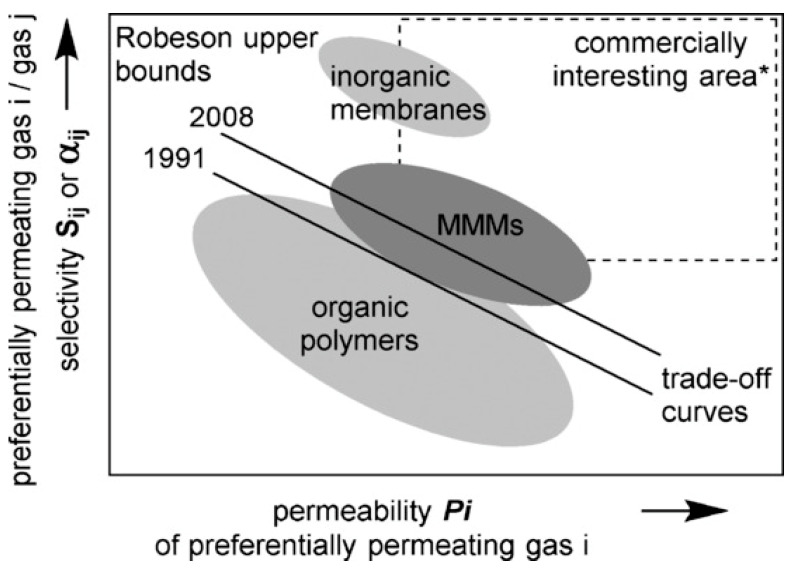
Robeson trade-off analysis selectivity and permeability in MMMs, inorganic, and polymeric membranes Reprinted/adapted with permission from Dechnik et al. [66] (2017) John Wiley and Sons.

The primary goal of inorganic filler incorporation into the polymer matrix is to overcome the Robeson upper bound, which is related to the trade-off relationship between permeability and selectivity of different pairs of gases including CO_2_/CH_4_, O_2_/N_2_, H_2_/N_2_, H_2_/CH_4_, He/N_2_, He/CH_4_, H_2_/CO_2_, He/H_2_, and He/CO_2_ [64]. It was plotted using a log–log plot of pure gas permselectivity (*P_i_*/*P_j_*) versus *P_i_* where *P* represents the polymer permeability, and *i* represents the more permeable gas pair *i* and *j* [65]. As illustrated in Figure 4, inorganic membranes and MMMs can overcome the selectivity and permeability trade-off, attracting development for commercial purposes. With the limitation of an inorganic membrane in terms of economics, the approach of MMMs will be much more prominent in the future [64,66]

Inorganic nanoparticles in a polymer matrix work as barriers for gas molecules, reducing gas penetration through membranes and aiding in preventing aging and plasticization [67]. They also have a high potential for adsorption of CO_2_ because of their intrinsic affinities.

To provide high-performance MMMs, material selection is one of the crucial factors in fabricating membranes. The morphology of membranes is closely related to the types of material and independent of the method of synthesis used. Additionally, the membrane separation process’s efficiency is dependent on the chemical interaction between the membrane material and the gas penetrant [68]. The properties of each material contribute to the membrane performance and structural properties. Material selection criteria include high separation efficiency with mechanical and thermal stability, chemical resistance, reasonable flux, and low cost [69]. When selecting inorganic materials, highly selective polymers can improve the separation performance of mixed-matrix membranes. Hence, in most studies, glassy polymers were chosen in developing mixed-matrix membranes over rubbery polymers due to their high selectivity properties. However, glassy polymer membranes are characterized by a weak organic–inorganic interaction, which results in voids at the polymer–filler interface. Thus, it is necessary to consider the gas separation properties and the adhesion of the two phases. In some studies, the glass polymer was blended with rubbery polymer to improve the polymer matrix structure.

Additionally, fillers’ interaction with polymers and textural characteristics and selecting appropriate inorganic filler materials are other criteria considered when fabricating mixed-matrix membranes [61]. Non-selective holes may form if the adhesion between the particles and the polymer matrix is inadequate, resulting in a high permeability of all gases and a subsequent loss of selectivity. Additionally, they may affect the mechanical properties [70]. On the other hand, they may stiffen the polymer chains surrounding the particles, closing the fillers’ selective holes and lowering permeability and selectivity. Numerous material combinations have been investigated to fabricate defect-free membranes capable of exceeding the upper bound limit.

Porous and non-porous inorganic materials are used in MMMs. Both are composed of distinct structures, which results in distinct mixed-matrix structures and pore sizes. Due to their pore size distribution and surface chemistry, porous filler materials such as graphene oxide, zeolite, CMS, and MOFs have higher compatibility with the polymer matrix than nonporous fillers, resulting in enhanced gas separation performance. Additionally, they act as molecule-sieving agents within the polymer matrix, separating molecules according to their shape and size.

MOFs have excellent wetting properties between two phases due to their organic moiety [71]. Among them, zeolitic imidazole frameworks (ZIFs) have garnered considerable interest for their superior chemical and thermal stability. Tao Li et al. [38] used surface coating to incorporate ZIF-7 nanoparticles into the Pebax 1657 polymer matrix. The membranes’ morphology demonstrates an extreme adhesion between the two phases (Pebax 1657 and ZIF-7). The inclusion of ZIF-7 filler increased the selectivity and permeability for CO_2_/CH_4_ up to 22 wt.%. However, as the loading increases, the permeability decreases due to the rigidity of the polymer chain. Jomekian et al. [72] introduced a co-casting method to fabricate a thin and defect-free selective layer. ZIF-8 was incorporated into Pebax 1657 polymer matrix and cast onto the PES sub-layer. The result indicates that CO_2_ has high permeability and a relatively constant selectivity at higher feed pressures, owing to increased CO_2_ sorption in the Pebax and ZIF-8 pore matrix. CMS has a microporous structure that enables the kinetic separation of gas mixtures and excellently separates gases such as CO_2_/CH_4_, O_2_/N_2_, CO_2_/N_2_, and [73]. Gases with smaller kinetic diameters, such as He and CO_2,_ are expected to easily permeate through the membranes compared with larger gases such as CH_4_, i.e., high selectivity.

Janshir et al. [74] synthesized composite mixed-matrix membranes (CMMMs) made of polyethersulfone (PES) and a carbon molecular sieve (CMS 1–5% wt.%) as an inorganic filler, as well as Novatex 2471 nonwoven fabric (support layer). The CO_2_/CH_4_ selectivity increased by about 109.73% compared to pristine PES. Due to the high electron density of aromatic rings on nanosheets, graphene oxide is known as an excellent inorganic filler. The separation mechanism depends on the formation of molecular sieving galleries between adjacent nanosheets or potential nanosheet defects. Guanying et al. fabricated MMMs using Pebax 1657 as a polymer matrix and porous-reduced graphene oxide (PRG) as inorganic fillers with ethanol–water (70/30 wt.%) mixture as a solvent. The crystallinity and rigidification of the polymer matrix at the Pebax-PRG interface increased, increasing CO_2_/N_2_ selectivity while decreasing the permeability. CO_2_ had the highest permeability of 119 barrer with a pressure of 0.2 MPa at 30 °C.

## 5. Carbon Nanotubes (CNTs)

**Figure 5:**
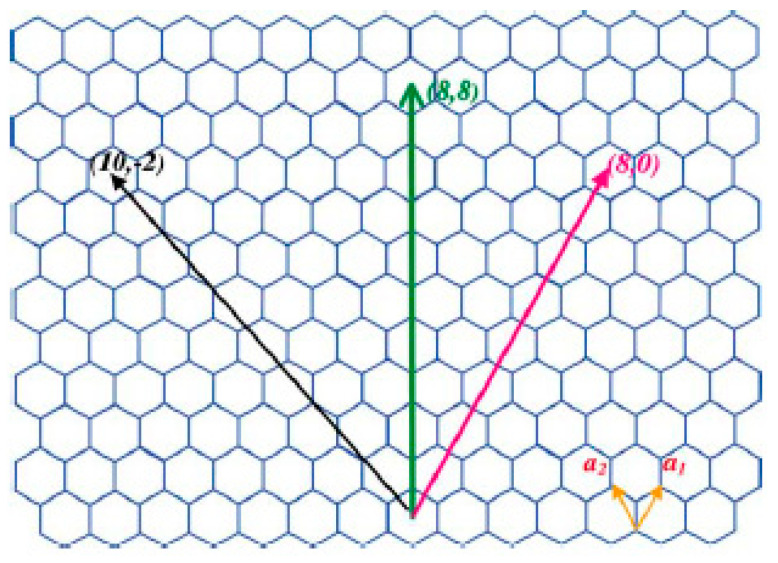
Illustration of graphene sheet’s honeycomb structure. Carbon atoms form the vertices. Folding the sheet in the direction of the lattice vectors can produce SWNTs. Reprinted/adapted with permission from Dai et al. [19] (2002) Elsevier Copyright.

Endo first synthesized carbon nanotubes (CNTs) in 1976 before Iijima worked on the detailed structure characterization in 1991 [75]. Iijima discovered multi-walled nanotubes (MWNTs) in carbon soot made by the arc-discharge method. Two years later, single-walled nanotubes (SWNTs) were discovered by Bethune and coworkers using the same method [76]. CNTs are divided into single-walled nanotubes (SWNTs) and multi-walled nanotubes (MWNTs). SWNTs consist of a graphene sheet rolled over into *sp*_2_ bonded carbon atoms with a diameter of about 1.4 nm, similar to a C_60_ bucky-ball [19,77]. Sometimes, the tubule diameter is too small and exhibits the effects of one-diameter (1D) periodicity. MWNT is a concentric cylinder with an interlayer spacing of 3.4 Å and a diameter typically around 10–20 nm (Figure 5).

**Figure 6:**
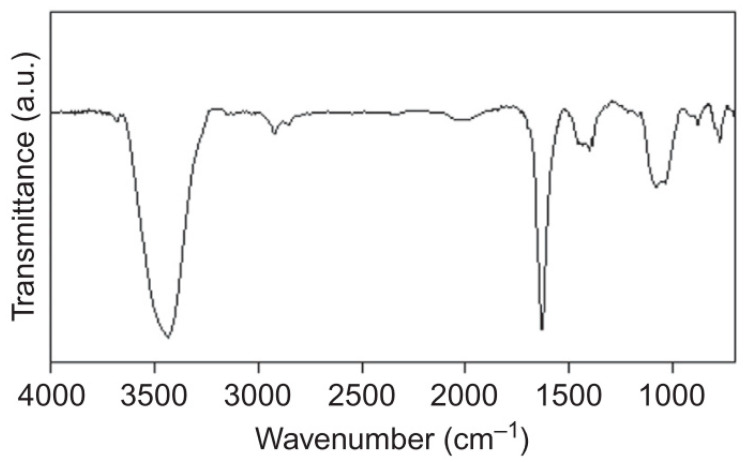
FTIR spectrum of raw CNT. Reprinted/adapted with permission from Ferreira et al. [79] (2019) Elsevier Copyright.

SWNT is formed when the beginning of the graphene sheet is folded over so that it touches the end of the (*m*,*n*) lattice vector, thus obtaining a (*m*,*n*) nanotube. Hence, the (*m*,*n*) indices determine the diameter and chirality of the nanotube. Both diameter and chirality of a nanotube are important in determining the properties of a nanotube, as a slightly different angle may change the properties of SWCNTs from a metal to a semiconductor. If the (*m*,*n*) has a difference of multiple three, the SWNT is said to be metallic; if the difference of (*m*,*n*) is not a multiple of 3, it possesses semi-conductor characteristics [78]. Unlike MWCNTs, the atoms of SWCNTs form a single covalently bound array. Because of this difference, SWCNTs have been used over MWCNTs in electric components due to their distinct electrical and optical properties. Figure 6 shows the spectrum of raw CNT to identify the functional groups in the materials. A sharp band spike can be observed around 1600 cm^−1^, which is related to C=C bonds of aromatic rings of the folded graphene, and the band along 1700–1600 cm^−1^ indicates the presence of O-H bending. A band at 3800–3200 cm^−1^ refers to O-H stretching. These bands correspond to water molecules absorbed from the ambient moisture or due to a purification process. Additionally, bands between 2910 and 2940 cm^−1^ are observed in the FT-IR spectrum of carbon nanotubes, which are associated with the C-H stretching vibrations of methylene (CH_2_) [79].

Properties of CNTs vary depending on the chirality in terms of the angle and vector of rolled-up directions. In terms of mechanical strength, CNTs with high flexibility and strength and high stiffness are known for their superiority over graphite fibers. This is because of the strongly bonded sp^2^ C=C and their high aspect ratio. The yield strength of CNTs was reported as ~0.64 TPa compared to steel at ~300 MPa, with CNTs being only one-sixth the weight of steel. Incorporating CNTs into the polymer matrix has been proven to increase mechanical strength. For instance, 1 wt. % of MWCNT addition to polystyrene/MWCNT nanocomposite films increased the break stress and tensile strength by about 25% and 40–42%, respectively [79]. Nonetheless, most studies reported a decrease in nanocomposites’ impact toughness, and only a few studies reported an increase in the impact toughness. A recent study confirmed that when 1 wt.% CNTs were incorporated with polyethylene, ductility and toughness increased by 104% and 150%, respectively [79].

CNTs incorporated in the polymer matrix show a desirable enhancement in thermal strength. The constraint effect on polymer segments and chains found in CNTs enables an upgrade in the glass transition temperature (*T_g_*) and the melting and thermal decomposition temperatures of the polymeric matrix. A study reported that the addition of 3 wt.% SWCNTs to epoxy increased its thermal conductivity up to 300% [79].

## 6. CNT-Polymer Nanocomposites

Due to the intrinsic smoothness of MWCNTs, CNTs in MMMs contribute to the highly rapid transport of gases and mechanical properties compared to other inorganic fillers [80]. Additionally, carbon nanotubes have significantly greater permeability and selectivity than any other recognized inorganic materials. When carbon nanotubes are combined with certain polymers, their permeability can be considerably enhanced by increasing their diffusion coefficients. CNT MMM development has accelerated significantly in recent years, particularly in gas separation [2,18] and water treatment [81,82]. The interfacial interactions between the carbon nanotubes and the polymer and their dispersion in the polymeric matrix affect the overall performance of CNT MMMs [83].

### 6.1. Dispersion of CNTs

Although carbon nanotubes have superior properties to other inorganic fillers, their applicability in mixed-matrix membranes for large-scale industries remains uncertain due to their inert chemical characteristics and incapability to disperse in typical organic solvents [52]. This is due to the properties of CNTs, which have very strong van der Waals forces between them. They tend to agglomerate and form tight bundles; hence, homogenous dispersion in the polymer matrix is difficult to obtain [19]. This has been the biggest challenge in developing CNT MMMs in producing defect-free membranes. The dispersion state of carbon nanotubes in various solvents is frequently used to better understand the carbon nanotube–solvent interaction and identify new approaches to improve their dispersion [79]. Improved dispersion of CNTs can be approached either by physical (ultrasonication, ball-milling, extrusion, and high shear mixing) or chemical methods. Chemical methods, also called CNT functionalization, include covalent and non-covalent methods.

#### 6.1.1. Covalent Functionalization

Covalent functionalization alters the translational symmetry of carbon nanotubes by modifying sp^2^ carbon atoms to sp^3^ carbon atoms [84]. There are two methods for functionalizing carbon nanotubes: modifying surface-bound carboxylic acid groups or directly attaching reagents to the side walls. During the oxidation process, functional groups such as –COOH, –OH, –F, and –NH_2_ are formed on the surface of carbon nanotubes. The most frequently used method is to treat the surface with strong inorganic acids. The functional groups on the surface of the carbon nanotubes were attached during the treatment to improve their compatibility with the polymer matrix [85]. Due to the attachment, the hydrophobic nature of carbon nanotubes is diminished, and they become hydrophilic, allowing for homogeneous dispersion of the functionalized MWCNTs in a broad range of organic solvents.

Ghaemi et al. [86] used phase inversion induced by immersion precipitation to prepare amine-functionalized MWCNTs/PES membranes. Chemically, pristine MWCNTs were treated with a mixed acid (sulfuric acid (H_2_SO_4_) and nitric acid (HNO_3_)) solution at a 3:1 volume ratio. Before filtering, the solution was stirred for 1 h at 90 °C. The carboxylated MWCNTs were dried for 1 h in a vacuum oven at 100 °C. They were then added to a mixture of 1,3-phenylenediamine (mPDA) and DMF solvent and stirred for 96 h at 70 degrees Celsius. SEM analysis revealed that amine-functionalized MWCNTs had thicker structures than pristine MWCNTs, particularly at the end caps of CNTs, increasing membrane hydrophilicity. The addition of functionalized MWCNTs significantly increased the membrane’s surface hydrophilicity. Additionally, FTIR demonstrated the presence of amine functional groups on the surface of MWCNTs. They explained that incorporating f-MWCNTs into PES membranes improved the performance and antifouling properties. Covalent functionalization has been proven to increase the solubility and dispersion of CNTs in the polymer matrix. Nonetheless, this technique that requires alteration of structural properties of CNTs during sonication, oxidation, and acid treatment may impede the full potential of CNTs. Thus, non-covalent functionalization is an alternative to functionalized CNTs without forming chemical bonds.

Amirkhani et al. [87] grafted functional groups (–COOH, –NCO, and –H_2_) on the surface of MWCNTs, which were then incorporated into PEBAX matrix at 0.1–1 wt.% loading. Here, 4,40-diphenylmethane diisocyanate (MDI) was used to functionalized MWCNTs into MWCNT–COOH and MWCNT–NCO, and MWCNT–H_2_ was synthesized by adding distilled water to the synthesized MWCNT–NCO. The optimal performance of permeability and selectivity differed for various functional groups based on the loading conditions with MWCNT–COOH at 0.75 wt.% loading, MWCNT–NCO (0.3 wt.%), and MWCNT–H_2_ (0.5 wt.%). The performance of membranes improved with increasing loading until it reached the optimum point, after which the addition of MWCNTs caused filler agglomeration and poor performance. MWCNT–NCO had the highest permeability for all gases with CO_2_ at 148.86 barrer, followed by CH_4_ (5.14) and N_2_ (1.42). Incorporation of functionalized groups disrupted polymer chain links and affected interfacial interactions and chain mobility, thereby affecting the fractional free volume (FFV) and polymer density. It has been demonstrated that -NCO groups enhance the inter-chain interaction of MWCNT–NCO membranes with the highest *T_g_* relative to other membranes, resulting in high permeability and selectivity.

Singh et al. [88] prepared PSF-based MMMs incorporating PEG-grafted CNTs as inorganic fillers with loading varying from 2.5–7.5 wt.% via initial solvothermal mixing followed by solvent casting. A uniform dispersion achieved up to a certain filler loading contributed to the high permeability result. Moreover, MMMs demonstrated a 12.5% increase in mechanical strength compared to pure PSF.

#### 6.1.2. Non-Covalent Functionalization

Non-covalent functionalization has no detrimental effect on the properties of carbon nanotubes because it involves the adsorption of chemical moieties onto the surface wall of the carbon nanotubes. As a result, it does not affect the final properties of carbon nanotubes. This method utilizes surfactants, biomacromolecules, or polymer wrapping. Hydrophobic micelle components surround the nanotubes. When the hydrophobic portion of the amphiphilic consists an aromatic group, the interaction becomes stronger.

The specific reaction between polymers and CNTs is the wrapping mechanism [84]. Mousavi et al. [89] fabricated PEBAX-1657/chitosan-wrapped MWCNTs on an ultra-porous polyethersulfone (PES) substrate. The MWCNTs were functionalized using carbohydrate polymer chitosan using the simple-mixing method. Chitosan was dissolved in acetic acid/water (2:98%). Polymers that wrap around CNTs are also known as supramolecular complexes. In this case, π-stacking interaction between the polymer and nanotubes surface is responsible for the close association of the structures. The addition of chitosan increases the adsorption capacity of the membrane, thereby increasing its antifouling property. Additionally, SEM images demonstrated a well-coated PEBA layer on a porous PES support and uniform dispersion of f-MWCNTs within the PEBA matrix.

Rajashree et al. [90] wrapped carbon nanotubes with carboxymethyl chitosan (CMC) via wet grinding-assisted sonication. The nondestructive walls of CNTs observed in FETEM analysis indicated the polymer wrapping did not affect the CNTs’ integral architecture. Raman spectroscopy analysis also suggested that the G-band shifted to a greater wavenumber of 1590 cm^−1^ from 1575 cm^−1^, subsequently wrapping with CMC, whereas the D-band had a negligible spectral shift. The intact position observed in the D-band suggested that CMC had not bonded covalently to CNTs. The increase in the G-band wave number resulted from the field disturbance caused by the CMC coating on the CNTs. This indicates that CMC and CNTs interact strongly.

A non-wrapping mechanism is another approach to non-covalent functionalization of CNTs. In this case, copolymers are introduced as the stabilizers to disperse CNTs in the solvents. Fernandes et al. [91] suggested that the triblock copolymer, Pluronics F127, adsorbed to CNTs via a non-wrapping mechanism and a central hydrophobic polypropylene oxide block flanked by hydrophilic polyethylene oxide blocks acted as the physical barrier to form a stable dispersion of SWNTs and MWNTs. Nonetheless, the non-covalent attachment of molecules may be weak, which will lead to the low efficiency of carbon nanotubes loading into the polymer matrix.

### 6.2. CNT–Polymer Mixed-Matrix Membrane in Gas Separation

CNT MMMs can be applied in various gas separation processes, including the separation of carbon dioxide, oxygen–nitrogen separation, and hydrogen separation. Hussain et al. [92] incorporated MWCNTs into polymer-blended CA/PEG membranes with 5–15 wt.% loading. Membranes with a loading of 10 wt.% had the best dispersion of filler particles and polar ether groups, resulting in increased crystallinity. The molecular sieving property of filler particles allowed for the highest CO_2_/CH_4_ selectivity. At higher loading of CNTs, agglomeration of CNTs occurred, creating non-selective voids between fillers and the polymer matrix. The addition of MWCNTs increased the thermal stability of membranes, allowing a 290 °C operating temperature, making it industrially very useful where separation occurs at higher temperatures. Akshay et al. [93] developed a hollow fiber membrane using PES and carboxylated CNTs. The membrane showed excellent mechanical strength; Young’s modulus increased from 268.1 ± 4.1 MPa for pristine PES to 409.1 ± 4.5 MPa for PES–CNT membranes. This resulted from the enhanced interfacial compatibility between oxygen-containing functional groups on carboxylate CNTs and sulfone groups on the PES matrix.

Yousef et al. [94] prepared PES/CNT membranes and recorded the separation performance of CO_2_, CH_4_, N_2_, and H_2_ gases. These authors varied the CNT loading (0.01–0.03 wt.%), pressure (1–6 bar) and temperature (20, 40, and 60 °C). N_2_ recorded the highest permeability at 10.5–15.4 barrer followed by H_2_ (8.4–12.1 barrer), CO_2_ (8.8–14.2 barrer), and CH_4_ (3.4–5.6 barrer). Permeability increased as the temperature increased due to the plasticization effect on PES. The behavior and character of the gases changed with the applied heat. The membrane loaded with 0.02 wt.% carbon nanotubes had a lower permeability than 0.01 and 0.03 wt.% carbon nanotubes. This was due to the uniform distribution of carbon nanotubes in the sample, which increased the matrix’s crystallinity degree by partially aligning the molecular chains and forming lamellae regions, obstructing gas transport through the membrane. Membranes with 0.01 and 0.03 wt.% CNTs loading had a random distribution of CNTs, decreasing the crystallinity degree and disrupting the PES chains, thus having a better permeability. Selectivity values of CO_2_/CH_4_ and CO_2_/N_2_ were 1.62 and 0.87, respectively.

Yu et al. [95] used carbon nanotubes to reinforce Pebax-1657 polymer membranes. The CO_2_ permeability increased as the CNT content increased and reached a maximum of 5% wt CNTs. The increased gas permeability explained the increased gas diffusivity of carbon nanotubes. The mechanical strength of membranes was determined, and the tensile modulus increased by 43% when 5 wt.% single-wall carbon nanotubes (SWNTs) were added and by 24% when 5 wt.% multi-wall carbon nanotubes were added (MWNTs).

Dai et al. [96] investigated the CO_2_ separation properties of Pebax/carbon nanotube–polyethylene glycol hybrid membranes. CO_2_ permeability was 369.1 barrer with CO_2_/N_2_ selectivity of 110.8 for a hybrid membrane containing 3 weight percent CNT–PEG, surpassing the CO_2_/N_2_ Robeson upper limit. Lee et al. [97] used the wet phase inversion approach to integrate dispersant-functionalized multiwalled carbon nanotubes (MWCNTs–F) into a polymer matrix of varied molecular weight (70,000 with 12,000, 30,000, and 65,000 Mn). CO_2_ had a selectivity of 17.09 for N_2_ and permeance of 341.15 for CO_2_.

Shin et al. [98] studied the performance of 0.1 wt.% functionalized MWCNTs incorporated with 4 wt.% cellulose acetate butyrate (CAB), and the effect of different loadings from 0.2–1.2 wt.% was observed. The membrane was evaluated using a CO_2_ and N_2_ single permeation test. The findings indicated that the MMM composed of CAB polymer and 0.1 wt.% MWCNTs performed better in CO_2_/N_2_ selectivity, with a value of 2.887. Farid et al. [87] grafted several functional groups (–COOH, –NCO, and –NH2) onto the surface of MWCNTs before incorporating them into a poly(ether-block-amide) (PEBAX) polymeric matrix. MMMs’ CO_2_ permeability and ideal CO_2_/N_2_ and CO_2_/CH_4_ selectivity were compared to the plain membrane. The findings indicated a significant increase and exceeded the CO_2_/N_2_ Robeson upper limit under 4–10 bar in the temperature range of 15–55 °C. MMMs with all three kinds of functional groups exhibited increased CO_2_ permeability and CO_2_/N_2_ and CO_2_/CH_4_ selectivity compared to pristine MWCNTs, demonstrating the strong adherence of functionalized MWCNTs to the polymer matrix.

The third component is an additive that can further enhance the performance of MMMs. Low-molecular-weight components such as diethanolamine (DEA), amine, ionic liquids, and chitosan are often used as the additive to promote homogenous dispersion of inorganic fillers in the polymer matrix. Murali et al. [45] prepared MMMs using Pebax-1657 and MWCNTs with the addition of 2,4-toluylene diisocyanate (TDI) to study the gas permeation properties of O_2_, H_2_, CO_2_, and N_2_ gases. The CNT loading was varied to study the effect of loading on the permeability of the membranes. The selectivity of a cross-linked 2% MWNT Pebax membrane for the CO_2_/N_2_ gas pair was increased from 83.2 to 162 with increasing input pressure (1–3 MPa). The incorporation of MWNT increased the free volume. The cross-linking with TDI reduced the polymer’s ion exchange capacity.

Moghadassi et al. [99] reported studies on functionalized carboxyl-MWCNTs incorporated with a polycarbonate (PC)/polyethylene glycol (PEG) polymer matrix at different loading ratios (1, 2, 5, and 10 wt.%). CO_2_ gas with a small kinetic diameter had the highest permeability result. For N_2_ and CH_4_ gases, the permeation process is similar to diffusion via the inner surface of carbon nanotubes. Hence, a higher loading rate of MWCNTs promoted the diffusion of the gases. As a result, the selectivity of CO_2_/CH_4_ and N_2_/CH_4_ increased as the loading rate of MWCNTs increased. Selectivity started to show a decrease at a loading rate 5 wt.% due to the trade-off of rapid diffusion of large gas molecules. The highest CO_2_/CH_4_ selectivity at 2 bar pressure and 25 °C was 27.38 for 5 wt.% of functionalized carboxyl–MWCNTs. Moghadassi et al. [100] also studied a cellulose acetate (CA)/styrene-butadiene rubber (SBR) blend polymer with both raw and functionalized MWCNT mixed-matrix membranes for the same types of gases. Permeabilities of gases increased with the increase of the CNT loading ratio. Some CNTs act like pinholes when vertically aligned to the membrane surface and create chances for diffusion mechanisms. The number of pinholes increases as the loading ratio increases, resulting in increased permeability. The increase in MWCNTs also increases FFV due to solution heterogeneity, creating voids between polymer chains and MWCNTs. The interaction between modified CNTs and polymer chains creates an interfacial zone that increases the relative sorption of gases; hence, permeability increases. However, the permeability was constant when the modified CNT loading ratio was higher than 0.65 wt.% due to adsorption stopping. Carboxylic groups that form on the surface of the filler material may act as a barrier to the entry of the nanotubes.

Dilshad et al. [101] studied the impacts of pressure on cross-linked polyvinyl alcohol (PVA)/polyethylene glycol (PEG) membranes tethered with surface-engineered multiwalled carbon nanotubes (SE-MWCNTs). The permeation of pure CO_2_, N_2_, and CH_4_ was recorded. They discovered that a membrane-tethered with 0.5 wt.% SE-MWCNTs did not exhibit an abrupt increase in permeability and a significant decrease in gas selectivity up to 20 bar pressures, demonstrating that the membrane was not plasticized under mixed gas circumstances or at high pressure. At higher loading of SE-MWCNTs with 0.75 wt.%, the permeability and selectivity of all gases decreased sharply due to the occurrence of interfacial voids. This occurred due to MWCNTs agglomerating at higher loads due to the Van der Waals interaction forming dense bundles with an uneven orientation. The performances of these membranes are tabulated in Table 5 to highlight the permeability and selectivity of membranes under different parameters.

## 7. CNT MMMs Gas Separation Application

CNT is one of the interesting components of an inorganic filler in membrane technology. As mentioned above, some innovative approaches to CNT MMMs should be assessed or improved to maximize the full potential of CNTs. In addition, coupling membrane separation with other assistant methods, including adsorption, catalysis, and electro-chemistry, is worthy of further investigation [17]. CNT MMMs have demonstrated great promise in gas separation; consequently, they have been explored as a potential approach. With a good understanding of the behavior and characteristics of CNT MMMs, the performance of membranes in terms of permeability and selectivity shall increase rapidly.

One application of CNT MMMs is natural gas purification [103]. Natural gas consists mainly of CO_2_ as its impurity and needs to be removed to meet the pipeline specifications. Commonly, in the last few decades, amine absorption technologies have been preferred due to their high selectivity, up to 99.0 %. However, this technology requires high capital and operation costs and large floor areas. Nonetheless, in recent years, membrane technologies have started penetrating the market as the performance of membranes has improved over time. CO_2_ gas possesses condensability and quadrupole moments, making it plausible to use as a selective surface flow or adsorption as its separating mechanism for CO_2_/CH_4_ separation. According to molecular dynamics simulations, the gas penetration rates inside the one-dimensional pores of carbon nanotubes are orders of magnitude greater than those in any other known microporous material [104]. As a result, one-dimensional nanochannels made of carbon nanotubes can serve as CO_2_ transportation highways, accelerating CO_2_ permeation across the membrane.

Cheng et al. [105] fabricated SWNTs with triptycene-containing polyimide with various loading of nanotubes from 2–15 wt.%. Here, a polyimide containing hierarchical triptycene units (6FDA-TP) was utilized. The triptycene units may introduce π−π stacking and nanostructures shape-fitting interactions with CNTs, thereby facilitating the configuration of desirable interface morphology in composite membranes. They showed significant improvement in CO_2_ with 144 barrer, a 144% increase compared to membranes without CNTs. Recognizing the trade-off between permeability and selectivity, there was a slight decrease in selectivity, but it remained within the acceptable range. Dan Zhao et al. [103] prepared MMMs based on Pebax-1657 and carbon nanotubes (CNTs) via the solution-casting method. Glycerol triacetate (GTA) was added as the third component, which enhanced the solubility coefficient. It was reported that the gas permeability of MMMs increased linearly with the addition of CNTs. This might be because carbon nanotubes carry gas more quickly than any other known material owing to their intrinsic smoothness. Moreover, with the addition of GTA as an additive, the permeabilities of CO_2_ increased significantly. The highest achieved permeability was at 1408 barrer, with the highest concentration of GTA 10 times higher than that of pristine Pebax.

## 8. Conclusions

Mixed-matrix membranes dominate the research field of membrane technology because they have the potential to provide superior performance and surpass the Robeson upper boundary while also being economically viable. The material selection process is critical in defining the thermal, chemical, and mechanical qualities and the performance of membranes. This review focuses on incorporating CNTs in mixed-matrix membranes for gas separation. Due to the intrinsic smoothness of MWCNTs, CNTs exhibit superior features such as strong mechanical strength and rapid gas movement compared to other inorganic fillers. Nonetheless, the primary difficulty in producing CNT MMM is dispersing the carbon nanotubes in the polymer matrix owing to the high van der Waals interactions. There are numerous methods for resolving the problem, including physical and chemical methods. The most often used approach is carbon nanotubes’ covalent or non-covalent functionalization. Research on CNT MMMs was summarized, focusing on the dispersion and functionalization of carbon nanotubes and the fabrication technique for CNT MMMs. Additionally, the permeability and selectivity of these CNT MMMs have been discussed.

## Figures and Tables

**Table 1:** Advantages and limitations of separation technologies.

Technology	Advantages	Limitation	Refs.
Absorption	- Does not have a pretreatment process - Has recovery rates of up to 95% - Has product purity up to 99% volume.	- Requires high costs - Need to regenerate solvent, and the process has a high energy demand - It requires a large floor area and is not suitable for offshore application	[4]
Adsorption	- No solvent - Has better stability for feed with high impurity concentrations - Recovers CO_2_ concentration higher than 90 vol%	- Low solid-to-gas capacity - Low solvent regeneration rate - Requires a large floor area	[5,6]
Cryogenic	- Achieves more than 99% of CO_2_ capture at -150 °C operating temperature - Produces liquified CO_2_ for more accessible storage	- High operating cost - Need to operate at high pressure to prevent CO_2_ sublimation - Requires a large floor area	[7,8]
Membranes	- Simplicity - Requires minimum supervision - Small floor area requirement - Bulk removal	- Moderate purity compared to other technologies - Possible recompression of permeate - Possible plugging of impurities in the gas stream.	[7]

**Table 3:** Permeability and Selectivity of Polymer Membranes.

Membranes	Permeability (Barrer)					Selectivity					Refs.
	CO_2_	O_2_	N_2_	CH_4_	H_2_	CO_2_/CH_4_	CO_2_/N_2_	O_2_/N_2_	H_2_/N_2_	H_2_/CO_2_	
Glassy Polymer Membranes											
PSF	39				139		3.6				[43]
PES	10		12	4	10	2.5	0.8		0.8	1	[29]
Matrimid 5218	9.54		0.7	0.32	30.3	94.6			43.2	3.2	[28]
Cellulose Acetate	15.56		1.77	1.45		10.7	8.8				[44]
Rubbery Polymer Membranes											
Pebax	55.85	4.69	1.39		32.11		40.18	3.37	23.1	0.57	[45]
PIM-EA(H_2_)-TB	1391		53.1	62.6		22.22	26.20				[40]
PVDF	2.11			0.08		26.37					[41]
Polymer Blend Membranes											
PVA/PEG	52.9		2.03				26				[37]
Matrimid/PIM-EA(H_2_)-TB	198		6.83	9.1		21.66	28.99				[40]
Matrimid/PVDF	9.42			0.08		42.81					[41]

**Table 5:** Different MMMs’ permeability and selectivity in gas separation.

Membranes	Pressure (Bar)	Loading Ratio (wt.%)	Permeability				Selectivity		Refs.
			CO_2_	N_2_	CH_4_	H_2_	CO_2_/CH_4_	CO_2_/N_2_	
PES/MWCNT	2	1	3.2	0.15				22	[2]
	2	3	3.5	0.17				21	
	2	5	4.5	0.21				21	
	2	10	3.5	0.19				18.5	
Matrimid/MWCNT	2	2	13	0.84	0.81		16	15.5	[41]
	2	5	15	1	1		15	15	
	2	8	18	1.29	1.38		13	14	
	2	10	11	0.85	0.92		12	13	
PEBAX/MWCNT with TDI	1	2	3.54	0.03		2.51		83.2	[45]
	1	5	17.47	0.21		7.18		84.5	
PEBAX/MWCNT–NH_2_ with GTA	20	1	1408			213			[102]
PEBAX-MWCNT crosslinked	10	2							
	10	5							
PEBAX/CNT–COOH	10	0.75	132.30	1.55	5.47		24.18	85.32	[87]
PEBAX/CNT–NCO	10	0.3	148.86	1.42	5.14		28.95	104.92	
PEBAX/CNT–NH_2_	10	0.5	139.52	1.46	5.31		26.28	95.62	
PC-PEG/ MWCNT–COOH	2	1	8.35	0.18	0.28		25.73	28.19	[99]
PC-PEG/MWCNT–COOH	2	2	12.53	0.26	0.37		26.59	27.45	
	2	5	15.47	0.31	0.46		27.38	25.42	
	2	10	20.32	0.39	0.57		27.28	25.37	
PVA-PEG/MWCNT	1	0.5	115.57	0.57	1.41		82.26	202.75	[101]
	5	0.5	107.78	0.55	1.38		77.88	195.96	
	10	0.5	104.5	0.54	1.35		77.35	193.52	
	15	0.5	101.12	0.52	1.32		76.49	194.46	
	20	0.5	99.62	0.51	1.33		76.45	195.33	

## Data Availability

Not applicable.
